# Effects of Probiotics Supplementation on the Intestinal Metabolites, Muscle Fiber Properties, and Meat Quality of Sunit Lamb

**DOI:** 10.3390/ani13040762

**Published:** 2023-02-20

**Authors:** Ting Liu, Yanping Bai, Chenlei Wang, Taiwu Zhang, Rina Su, Bohui Wang, Yan Duan, Lina Sun, Ye Jin, Lin Su

**Affiliations:** 1 College of Food Science and Engineering, Inner Mongolia Agricultural University, Hohhot 010018, China; 2 Integrative Research Base of Beef and Lamb Processing Technology, Ministry of Agriculture and Rural Affairs of the People’s Republic of China, Hohhot 010018, China; 3 Inner Mongolia Vocational College of Chemical Engineering, Hohhot 010017, China; 4 Ordos City Inspection and Testing Center, Ordos 017000, China

**Keywords:** probiotics, intestinal metabolites, muscle fiber characteristics, meat quality

## Abstract

**Simple Summary:**

The meat quality of small ruminants is adversely affected when the feeding mode is changed from grazing to captivity. We found that adding probiotics to lamb feed regulated the intestinal metabolites (SCFAs) of Sunit lambs, acting as signaling factors that affected the gene mRNA expression of MyHC isoforms and the activity of metabolic enzymes, thereby promoting the transformation of muscle fiber types and increasing the proportion of oxidative muscle fibers. Finally, the tenderness of the meat was improved.

**Abstract:**

The development of animal husbandry is closely related to the meat quality of small ruminants. Intestinal metabolites and the muscle fiber types of lambs are important factors that affect their meat quality, but few studies have examined the regulation of the "intestinal muscle axis" by probiotics. In this study, 12 Sunit lambs were divided into a control group (C) and a probiotics group (P). The gene expressions of the myosin heavy chain, metabolic enzyme activity, and short-chain fatty acids in the intestines were analyzed using gas chromatography-mass spectrometry (GC-MS) and quantitative real-time PCR. The results showed that levels of propionic acid and butyric acid in the intestines of group P were significantly higher than in group C (*p* < 0.05). In addition, probiotics increased the number and area ratio of type I muscle fibers. They also increased the mRNA expression of MyHC IIA and the activity of malate dehydrogenase (MDH) and succinate dehydrogenase (SDH). Propionic acid was negatively correlated with the number ratio of type IIB muscle fibers. Butyric acid was found to be significantly positively correlated with the number ratio of type IIA muscle fibers. Cooking loss, pH_24h_, and shear force decreased significantly in group P. In conclusion, intestinal metabolites (SCFAs) altered the activity of oxidative-myofibril-metabolizing enzymes and the expression of myosin heavy-chain type IIA, reduced the meat shear values, and improved meat tenderness. This study provides a new basis for improving the production and meat quality of small ruminants.

## 1. Introduction

Inner Mongolia is the largest production base of lamb products in China. Sunit lamb is a Mongolian lamb with a good flavor, high nutritional value, and high-quality meat that is popular among consumers [1]. In order to protect pasture resources and slow down problematic pasture degradation, herders are gradually changing their feeding methods from grazing to housed feeding. However, this change in feeding methods has been shown to degrade lamb quality [2]. Therefore, the problem of improving the meat quality of captive-bred lamb requires urgent attention. Meat quality is closely related to the development of skeletal muscle [3,4], and muscle fibers are the basic unit of skeletal muscle. Muscle fiber characteristics include the fiber type, diameter, cross-sectional area, and density; among these, the fiber type has a significant influence on other meat quality indicators, such as tenderness, shear force, water retention, color, and intramuscular fat [5,6].

Muscle fiber types can be classified according to myosin heavy chain isoforms (MyHC) as oxidative (type I, type IIA), enzymatic (type IIB), and intermediate (type IIX) [7]. Type I fibers mainly rely on the oxidative metabolism to produce ATP, whereas type IIX and type IIB fibers mainly produce ATP through glycolysis [8]. It has been found that the difference in the enzyme activities of different muscle fiber types determines the difference in muscle metabolic capacity [9]. Type I muscle fibers have high myoglobin content, high aerobic enzyme activity, and low glycogen content, whereas type II muscle fibers have higher glycogen content and high ATPase activity, so the glycolytic process mainly occurs in type II muscle fibers [10]. When the proportion of oxidative muscle fibers is high, the redness value, pH, tenderness, and water retention levels of the meat are higher, which indicates higher quality meat [11].

Feed supplements modulate the gut microbiota and endocrine function to influence intestinal metabolites and skeletal muscle phenotypes [12]. Lactobacillus, an alternative to antibiotics, is widely used in ruminants to provide them with the nutrients required for growth, to increase their growth performance and immune modulation, and to promote the conversion of muscle fiber types. *Lactobacillus-fed B. fragilis* mice showed higher muscle mass compared to the control group; in addition, the modulation of Atrogin-1 and MyoD by gut metabolites (SCFAs) attenuated muscle loss in control group mice [13]. Expression of PPARβ/δ is predominant in skeletal muscle and is involved in energy metabolism, mitochondrial biogenesis, and fiber-type conversion [14]. In an analysis that aimed to characterize the effect of KLF6 on the gene expression profile of bovine adipocytes, differentially expressed genes (DEGs) were found to be associated with the PPAR signaling pathway and to play a role in regulating the lipid metabolism in bovine adipocytes, in addition to improving meat tenderness; DEGs could therefore be used as candidate gene markers to aid selection for growth and carcass traits [15,16]. The main potential mechanisms of the microbiome regulation of muscle include the protein, energy, lipid, and glucose metabolisms, and mitochondrial function [17]. Intestinal metabolites mainly provide nutritional sources for the host; for example, SCFAs, including acetic acid, propionic acid, and butyric acid, promote intestinal bacteria to fulfill different physiological regulatory functions and also bind and interact with G-protein-coupled receptors on immune cells and endocrine cells, resulting in epigenetic changes [18,19]. Propionate addition to these metabolites, for example, could affect growth performance and skeletal-muscle fiber-type transformation in cattle [20,21]. Numerous studies have indirectly shown that the ingestion of lactic acid bacteria may affect skeletal muscle development and metabolic status [22,23]. These findings allow us to posit a relationship between muscle characteristics and intestinal flora metabolites. However, few studies have explored the relationship between intestinal metabolites and skeletal-muscle fiber-type transformations. As such, we conducted an experiment in Sunit lambs, providing a theoretical basis for livestock breeding production and an exploration of probiotic regulation of meat quality by assessing the correlation between intestinal differential metabolites and muscle-fiber-type transformation.

## 2. Materials and Methods

### 2.1. Animals and Diets

The animal procedure was approved by the Animal Protection and Use Committee of the Inner Mongolia Agricultural University (NND2021072) according to the “Guide to Laboratory Animals” (GB 14925-2010) of the Ministry of Science and Technology Implementation. A total of twelve three-month-old healthy Sunit lambs weighing 19.77 ± 4.05 kg were randomly selected from the Chuanjing Sumu Haratu Gacha area in the Urad Middle Banner of Bayannaoer City, Inner Mongolia, and divided into two feeding groups (6 lambs in each group; half of them male and half female). After three months of feeding, the final weight of the Sunit lambs in group C was 31.03 ± 2.83 kg, and that of the lambs in group P was 34.13 ± 5.65 kg. The animals were kept in 2 pens (both 10 × 8 m in size), and they were allowed to move freely and drink water that did not contain antibiotics during the experimental period. Lambs were fed a daily basic diet (0.5 kg concentrate and 0.45 kg corn). The basic diet was a typical corn–soybean meal diet without any antibiotics or drugs. The concentrate (Inner Mongolia Chia Tai Co., Ltd., Inner Mongolia, China) consists of 60% corn, 18% sunflower meal, 13.4% soybean meal, 4% rapeseed meal, 1% stone powder, 0.5% calcium bicarbonate, 0.7% sodium chloride, 0.5% lysine, 1.2% calcium, 0.6% phosphorus, a vitamin and mineral premix, etc. Group C lambs were fed a basic diet, while group P lambs were fed a basic diet and probiotics. The probiotics were a compound of *Lactobacillus plantarum* HM-10 and *Lactobacillus casei* HM-09 (Inner Mongolia Hemeikesheng Biotechnology Co., Ltd., Inner Mongolia, China), with a viable count of 1.50 × 10^9^ cfu/g. The lambs were fed once a day, and the probiotics were added at a rate of 2 g per lamb.

### 2.2. Sample Collection at Slaughter

The Sunit lambs were taken off feed after 90 days and rested for 12 h before slaughter (Grassland Hengtong Food Co., Ltd.). After slaughter, the body was cut longitudinally into two halves (right and left) along the spine of the lamb. After debonding, the muscle longissimus thoracis (LT) on the left side of the 9th to 13th ribs was sampled and measured. The LT muscle was sectioned in the following order for the respective determinations: cutting from the caudal part of the LT towards the cranial part, a 2 cm thick piece for pH and color determination, a 1 cm thick piece for shear force, a 1 cm thick piece for histological observation of ATPase staining, a 0.5 cm thick piece for total RNA extraction, and a 100 g weight slice was used for cooking loss. Fecal samples were collected from the colons of twelve Sunit lambs and stored immediately at −80 °C until the determination of short-chain fatty acids.

### 2.3. Meat Quality Analysis

#### 2.3.1. Color

The LT muscle of each sheep was processed to 3 cm × 3 cm × 2 cm. The brightness value (L*), red value (a*), and yellow value (b*) of the LT muscle at the junction of the thoracolumbar spine were measured using a TC-P2A automatic colorimeter (Shanghai Biological and Biochemical Experimental Instrument Co., Ltd., Shanghai, China); the chromometer was calibrated with a standardized white tile at 2 °C observer angle, 50 mm aperture size, and the illuminant D65. The same sample was measured three times, and the average value was calculated.

#### 2.3.2. pH Value

The pH value of the LT muscle at the junction of the lambs’ thoracolumbar spines was measured 45 min after slaughter with a pH-STAR-type carcass pH meter (Matthaus Company, Erlangen, Germany) and recorded as pH_45min_. After standing for 24 h (4 °C), the pH value was measured again and recorded as pH_24h_. Two parallels were measured for each meat sample, and each parallel was measured twice. The deviation in the results was less than 5%, and the average value was taken.

#### 2.3.3. Cooking Loss

Approximately 100 g of the LT muscles at the junction of the thoracolumbar spine were sampled, and their mass was determined (*m*_1_). They were then placed in a sealed bag and cooked in a water bath at 80 °C. Once the central temperature of the meat sample reached 70 °C, it was removed from the water bath and cooled at room temperature; the surface water was dried using filter paper, and its mass (*m*_2_) was weighed. The cooking loss is calculated using the following formula:

$$cooking\;loss\;\left(\%\right)=\frac{m_{1}-m_{2}}{m_{1}}\times 100$$

#### 2.3.4. Shear Force

After discharging acid for 24 h, the LT muscle at the junction of the thoracolumbar spine was cooked in an 80 °C water bath for 45 min. Once cooled to room temperature, the sample was removed from the bath, and the surface was dried. The sample was trimmed into a 3 cm × 1 cm × 1 cm cuboid along the direction of the muscle fibers and measured using a C-LM3B digital display muscle tenderness meter (College of Engineering, Northeast Agricultural University). The same sample was measured three times, and the average value was calculated.

### 2.4. Histological Analysis

Within 45 min of slaughter, the LT muscle was cut into blocks of 0.5 cm × 0.5 cm × 1 cm along the direction of the muscle fibers, put into isopentane pre-cooled by liquid nitrogen, and dehydrated and dried for 30 s. It was then put into liquid nitrogen for quick freezing and finally placed in a −80 °C refrigerator. The muscle fiber samples were cut into 10 μm-thick cross-sections in a −25 °C frozen slicer; one well-cut muscle fiber sample was selected, and the prepared sections were stained using the ATPase (pH 4.60–4.65) staining method [24]. The following solutions were prepared: an acidic pre-incubation solution (5.4 g NaAc dissolved in 100 mL distilled water with a pH adjusted to 4.60–4.65 with glacial acetic acid), an alkaline pre-incubation solution (20 mL 0.1 mol/L sodium barbiturate, 20 mL 0.18 mol/L CaCl_2_, and 60 mL distilled water in a beaker; pH adjusted to 10.0 with 0.1 mol/L NaOH), and an incubation solution (20 mL of 0.1 mol/L sodium barbiturate, 10 mL of 0.18 mol/L CaCl_2_, and 70 mL of distilled water in a beaker; then, 0.15 g of adenosine-5′-triphosphate disodium salt was added and the pH was adjusted to 9.4 with 0.1 mol/L NaOH). The sections to be stained were placed in a dye bath and incubated for 5 min at 37 °C in the acidic pre-incubation solution. The sections were placed in a new dye bath and incubated for 30 s at room temperature with an alkaline pre-incubation solution, then again placed in a new bath and incubated for 30–45 min at 37 °C. The slices were washed three times with a 1% CaCl_2_ solution (3 min each time) and then with a 2% CoCl_2_ solution for 5 min. Then, the slices were washed twice with a 0.01 mol/L sodium barbiturate solution (5 min for the first wash and 1 min for the second). Finally, the slices were washed with distilled water for 1 min and stained with 1% (NH_4_)_2_S for 1 min.

After thoroughly washing the samples with water, the sections were sealed with neutral gum, and the morphology of the muscle tissue was observed [24]. Muscle fibers were photographed at 100× magnification using a light microscope (Leica DMI4000B, Leica, Wetzlar, Germany). When the data were measured for each relevant contour, image processing software (Leica Qwin V3, Leica, Wetzlar, Germany) was used to analyze the indicators of muscle fiber properties. The total number of muscle fibers was not less than 1000, and the measured muscle fibers were randomly selected based on the absence of tissue damage and freezing injury; they were defined as type I fibers, type IIA fibers, and type IIB fibers.

### 2.5. Muscle Enzyme Activity

The activities of succinate dehydrogenase (SDH), malate dehydrogenase (MDH), and lactate dehydrogenase (LDH) in the muscle were determined using an assay kit (Nanjing Jiancheng Institute of Bioengineering, Nanjing, China). The mechanical tissue disrupter was used to generate muscle homogenates. This operation requires around 100 mg of frozen muscle and 1 mL of PBS. After centrifugation (2500 r/min, 4 °C), the supernatants were decanted and saved for assays of the SDH, MDH, and LDH activities. The SDH, MDH, and LDH activities were expressed as U/mg proteins in skeletal muscle tissue.

### 2.6. Real-Time PCR

When extracting RNA from the samples, we used the TRIzol reagent kit (TaKaRa, Dalian) from the TaKaRa Company, closely following the steps outlined in the manufacturer’s instructions. After that, reverse transcription of RNA was performed. In brief, 2 μg of the extracted total RNA was taken, and the desired cDNA was obtained with the help of the PrimeScript RT reagent kit with a gDNA eraser (TaKaRa, Dalian, China). The expression levels were normalized to 18S ribosomal RNA (18S rRNA). The primers used for the target genes (MyHC I, MyHC IIA, MyHC IIX, and MyHC IIB) were designed with the aid of Primer 5.0 software (PREMIER Biosoft International, Palo Alto, CA, USA) and synthesized by Invitrogen Technologies (Shanghai, China). Quantitative real-time PCR was performed using an ABI Prism 7000 detection system with SYBR Green (Applied Biosystems, Foster City, CA, USA) in two steps. The reaction solution was prepared according to Table S1, and the genomic DNA was removed at 42 °C for 2 min. The reaction solution was prepared according to Table S2, and the reverse transcription operation was performed at 37 °C for 15 min and 85 °C for 5 s. The concentration of cDNA obtained after reverse transcription was 50 ng/uL. The real-time quantitative PCR reaction system was configured according to Table S3 and the quantitative kit (TB GreenTM Premix Ex TaqTM II) for real-time fluorescence quantification of target genes.

The primer sequences used are shown in Table 1. The specific arrangement of the thermal cycling program is as follows: First, it was pre-incubated at 95 °C for 30 s, for a total of 40 cycles. Each cycle was followed by denaturation at 95 °C for 5 s, followed by annealing at 57 °C for 30 s, and a final extension at 72 °C for 30 s. The expression of the 16S RNA gene for each species was used as an internal control. All the experimental sample analyses were run in triplicate. The gene expression data were calculated using the 2^−ΔΔCt^ method and expressed as the ratio of targeted gene expression to that of the 16S RNA housekeeping gene. 

### 2.7. Short-Chain Fatty Acids in the Intestine

Twelve colon fecal samples were weighed separately, with each weighing approximately 1.0 g. Dilution was performed with 2 mL of a 0.9% (m/m) sodium chloride solution. Subsequently, 0.5 mL of sulfuric acid was added to the dilution. The resulting dilution was kept cold in an ice box for 30 min before undergoing cryogenic centrifugation (4 °C, 10,000 rpm, 10 min). Then, 2 mL of diethyl ether was added to 1.0 mL of the centrifuged supernatant. It was vortexed for 2 min and centrifuged again for 10 min. After that, it was incubated at 4 °C for 30 min, and the resulting extract was stored at −20 °C for testing. The extract (1 μL) was injected into a TR-Wax-MS-fused silica capillary column (Thermo, 30 m × 0.25 mm × 0.25 μm film thickness) and injected into a Thermo 1300 gas chromatography system. The detection mode of the Thermo ISQ ion trap MS system comprises a full scan. The operating conditions of the column are as follows: First, the initial temperature was set to 100 °C and held for 30 s; second, the temperature was increased to 180 °C at a rate of 8 °C/min and maintained for 1 min after reaching the predetermined temperature. Then, the temperature was rapidly increased to 200 °C and maintained for 5 min. During the injection, nitrogen was used as the carrier, the flow rate was constant at 1.2 mL/min, and the split ratio was set at 10:1. When injecting 1 μL of the sample, it was ensured that the injection temperature was 250 °C, the ion source temperature was 250 °C, and the scanning mass range was between 40 and 450 m/z. Under these conditions, different SCFAs could be distinguished and identified by their unique retention times.

### 2.8. Statistical Analysis

Differences in the parameters studied were analyzed using a one-way analysis of variance (SPSS 27 ANOVA) to compare means using the least significant difference (LSD) procedure. *p* < 0.05 was considered statistically significant. The relationship between the parameters was evaluated by a Pearson correlation analysis. Origin (version 2021) and R language version 4.1.1 were used for mapping.

## 3. Results

### 3.1. Effects of Probiotics on the Meat Quality of Sunit Lambs

**Figure 1:**
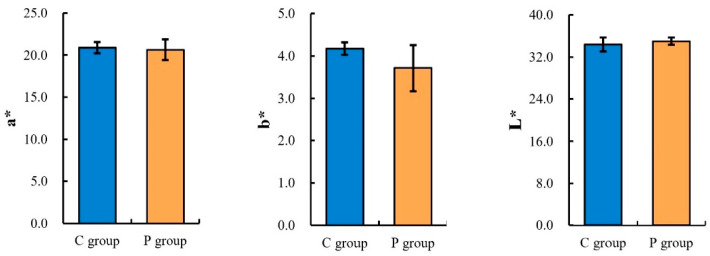

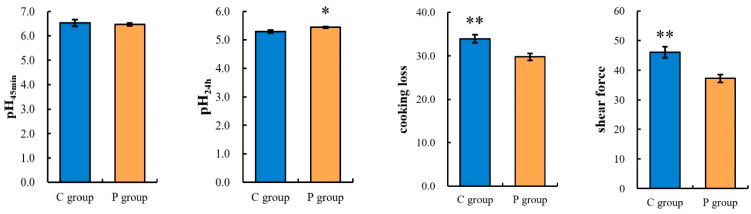
Effect of probiotics on the quality of Sunit lamb. * indicates a statistically significant difference (*p* < 0.05), and ** indicates a highly significant difference (*p* < 0.01). a* value means the redness, b* value means the yellowness, and L* value means the brightness.

The effects of probiotics on the meat quality of lambs are shown in Figure 1. The probiotics had no significant effect on meat color (a* value, b* value, and L* value), and there was no significant difference between the two groups in the pH_45min_ (*p* > 0.05). The pH_24h_ was significantly higher in the P group than in the C group (*p* < 0.05). Moreover, the cooking loss and shear force of the lamb in group P were significantly lower than those in group C (*p* < 0.01), which proves that the lambs fed probiotics produced more tender meat.

### 3.2. Effects of Probiotics on the Muscle Fiber Properties of Sunit Lambs

**Figure 2:**
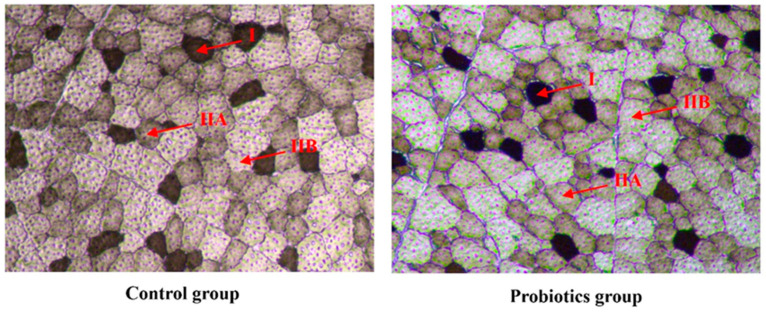
ATPase staining results for the muscle longissimus thoracis of Sunit lambs. A magnification of 200× was used (bar = 200 μm). I: fiber type I, black; IIA: fiber type IIA, white; IIB: fiber type IIB, brown.

The histochemical staining results of the ATPase of the muscle longissimus thoracis of Sunit lambs are shown in Figure 2. The number ratio, area ratio, diameter, cross-sectional area, and density of the muscle fibers were statistically analyzed as described before, and the results are shown in Table 2. Compared with the control group, the muscle fiber density of the probiotic group slightly increased after 90 days of feeding, but not significantly (*p* > 0.05). The oxidative muscle fibers (types I and IIA) accounted for more than 40% of the fibers. Moreover, probiotic supplementation significantly increased the number and area ratio of type I muscle fibers (*p* < 0.05). On the other hand, the cross-sectional area of type IIB muscle fibers tended to decrease, but the effect was not significant (*p* > 0.05). These results suggest that probiotics can increase oxidative muscle fiber (type I) content in the longissimus thoracis of Sunit lambs.

### 3.3. Effects of Probiotics on the Gene mRNA Expression of MyHC Isoforms

**Figure 3:**
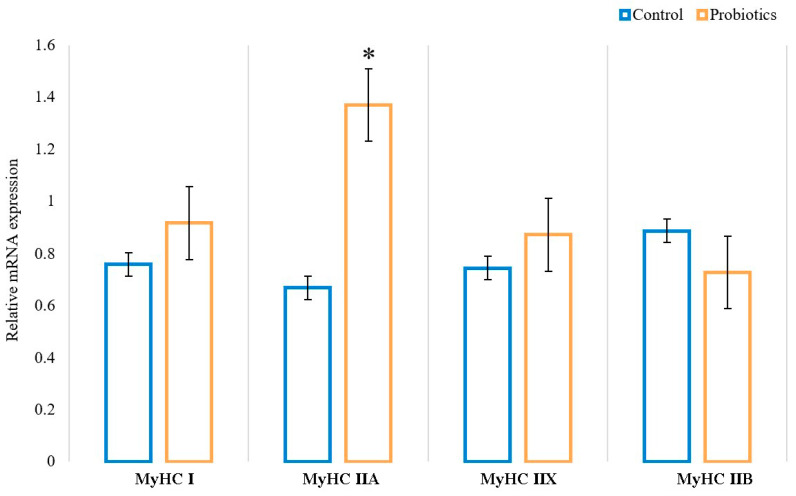
Effects of probiotics on the relative mRNA expression of myosin heavy chain (MyHC) isoforms (MyHC I, IIA, IIX, and IIB) in the muscle longissimus thoracis; 18S was used as an internal control. Data are expressed as means ± SE (*n* = 6). * indicates a statistically significant difference (*p* < 0.05).

As shown in Figure 3, the mRNA expression of oxidative fiber isoforms (MyHC I and IIA) was upregulated, and glycolytic fiber isoforms (MyHC IIB) were downregulated by dietary supplementation with probiotics in the longissimus thoracis of Sunit lambs. Specifically, the mRNA expression of MyHC IIA in group P was significantly higher than that in group C (*p* < 0.05). Additionally, dietary supplementation with probiotics had the effect of decreasing the gene expression of MyHC IIB, but there was no significant difference (*p* > 0.05). It was shown that dietary supplementation with probiotics can promote the transformation of oxidative muscle fibers.

### 3.4. Effects of Probiotics on Muscle Oxidative Metabolism

**Figure 4:**
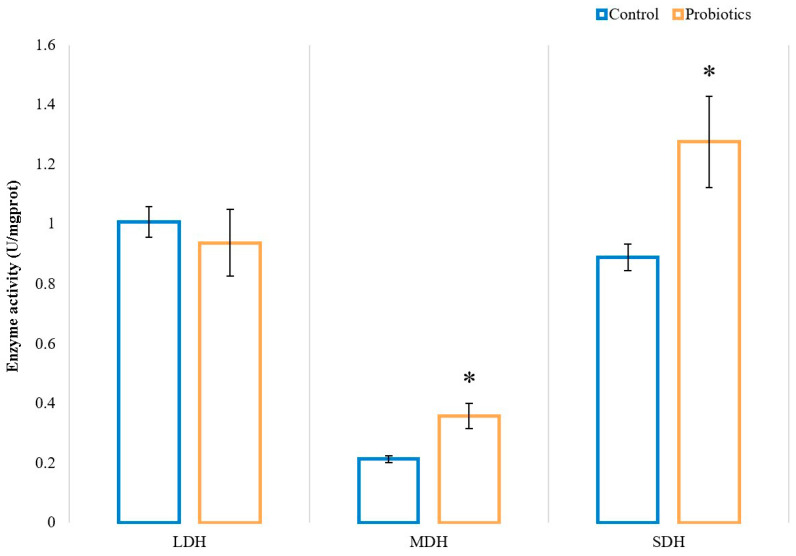
Effect of probiotics on the LDH, MDH, and SDH activities of the muscle longissimus thoracis. * indicates a statistically significant difference (*p* < 0.05).

We investigated the effects of probiotics on the activity of metabolic enzymes, and the results are shown in Figure 4. In the test group, both MDH and SDH activity were significantly increased (*p* < 0.05). However, there was no significant difference in the activity of LDH between the two groups (*p* > 0.05). These results suggest that dietary supplementation with probiotics contributes to increasing the oxidative metabolism level of muscles.

### 3.5. Effects of Probiotics on Intestinal Metabolites (Short-Chain Fatty Acids)

Colonic metabolites were examined in samples from Sunit lambs, and seven short-chain fatty acids were identified (Table 3). Butyric acid, valeric acid, and propionic acid were the metabolites found in the highest proportions in the colons of Sunit lambs, with values of 57.72%, 28.66%, and 27.51%, respectively. The concentrations of propionic acid, butyric acid, and valeric acid were significantly higher in group P than in group C (*p* < 0.05). The results showed that probiotics changed the composition of short-chain fatty acids in the colon of Sunit lambs.

### 3.6. Relationship between Intestinal Metabolites (SCFAs) and Muscle Fiber Types

**Figure 5:**
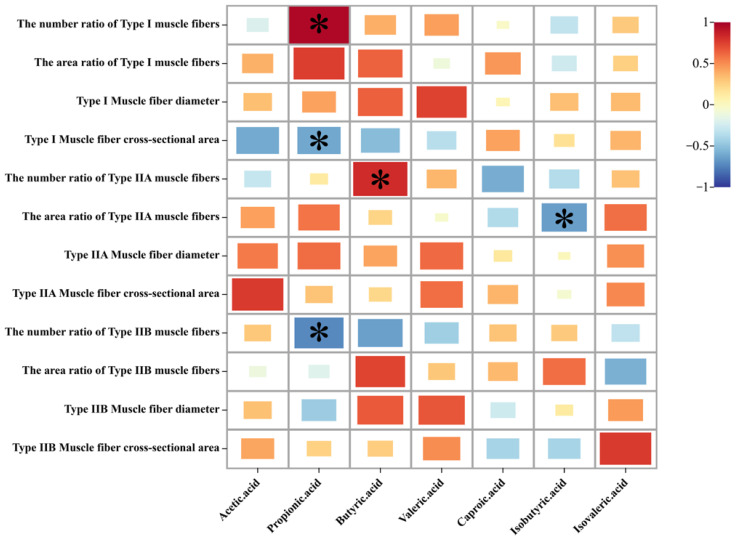
Correlation heat map of intestinal metabolites and muscle fiber types. The color band is mapped to the heat map matrix data; colors close to positive values indicate a positive correlation, while colors close to negative values indicate a negative correlation. * indicates a statistically significant difference (*p* < 0.05).

The above results indicate that dietary probiotics affected the intestinal metabolites (SCFAs) and muscle fiber types of Sunit lambs. From Figure 5, it can be seen that propionic acid was negatively correlated with the cross-sectional area of type I muscle fibers and the number ratio of type IIB muscle fibers but positively correlated with the number ratio of type I muscle fibers. Butyric acid was found to be significantly positively correlated with the number ratio of type IIA muscle fibers. Meanwhile, isobutyric acid was significantly negatively correlated with the area ratio of type IIA muscle fibers.

## 4. Discussion

Compared with grazing lambs, captive lambs produce lower-quality meat, and the probiotics that regulate meat quality are therefore worth studying. At present, the mechanism by which probiotics regulate meat quality is still unclear. This study found that probiotics significantly reduced the pH_24h_ value, cooking loss, and shear force value. Both cooking loss and shear force indicate the tenderness of the muscle. The lower the cooking loss, the higher the water-holding capacity of the muscle; similarly, the lower the shear force value, the greater the tenderness of the meat. Shear force is closely related to the growth of skeletal muscle and is crucial to the formation of good-quality meat. In other words, the number of oxidative muscle fibers is directly related to the tenderness, pH value, and water-holding capacity of muscle [25]. pH reflects the glycolytic rate of post-mortem muscle, which is related to the meat’s color and shelf life. The pH value of animals after slaughter was 6.8–7.3. A lower pH value can damage the muscle’s protein structure and adversely affect the meat’s quality. It was found that the percentage increase in type I fibers in pig longissimus muscle was related to the decrease in the pH value [26]. Therefore, the experiment started by examining the low-quality meat apparently caused by feeding with probiotics and explored the factors affecting the development of lambs’ muscle tissue after birth, which were reflected in changes to their muscle fiber cross-sections and diameter.

In this study, dietary probiotic supplementation increased the number and area ratio of type I oxidative muscle fibers, perhaps because the dietary nutrition level and intestinal absorption of nutrients affect the development of muscle tissue [27]. Elsewhere, mice on normal diets and high-fat diets were fed Bacteroides. The results showed that the muscle masses of both groups of mice increased, and the colonization and status of the intestinal bacteria were closely related to muscle growth and function [28]. According to the research conducted by Xiao Liu et al., microecological agents increased the diameter and cross-sectional area of pig muscle fibers and regulated the morphology of muscle tissue [29]. Increasing specific probiotics was not only conducive to increasing the average muscle fiber cross-sectional area of mice but also improved the muscle atrophy and muscle dysfunction caused by diseases [30].

The close relationship between intestinal flora and muscle tissue is defined as the “intestinal-microorganism–muscle axis” and the main substances at work in signal transmission are intestinal metabolites (SCFAs). Short-chain fatty acids are produced by beneficial bacteria in the microbiome. Carbohydrates produce a large number of SCFAs (such as acetate, propionate, butyrate, and valerate) after microbial fermentation in the intestine; these are the main media for intestinal bacteria to perform different physiological regulatory functions. SCFAs can directly affect the intestinal barrier and regulate intestinal movement. In addition, they can act as effector molecules, affecting muscle metabolism and function through the circulatory system [31,32,33]. In the next stages of the study, we will adjust the concentration of intestinal metabolites to affect the type of muscle fibers and lay the foundation for improving meat quality by reducing shear force and increasing tenderness. Studies have shown that butyrate supplementation could promote the production of oxidative fibers in the skeletal muscle of mice. According to the polymorphism of the myosin heavy chain (MyHC), muscle fibers can be divided into four types: MyHC I, MyHC IIA, MyHC IIX, and MyHC IIB. The increased expression levels of MyHC I and MyHC IIA in the gastrocnemius were associated with dietary sodium butyrate supplementation. The supplementation of sodium butyrate in feed could increase the content of myoglobin and troponin, which are muscle fiber factors associated with slow twitching. In vitro experiments showed that different doses of sodium butyrate could upregulate the mRNA expression of MyHC IIA and downregulate the mRNA expression of MyHC IIB in the C2C12 muscle cell line [34]. In addition, bacterial metabolites, such as conjugated linoleic acid and secondary bile acid, also affect the determination and transformation of muscle fiber types [35,36,37,38]. This experiment showed that the contents of propionic acid, butyric acid, and valeric acid in the intestine were higher after feeding with probiotics, and these short-chain fatty acids were significantly correlated with type I muscle fibers, type IIA muscle fibers, and type IIB muscle fibers.

Moreover, different muscle fiber types affect the body’s metabolism [39,40]. Muscle metabolism is related to the composition and type of muscle fiber. Type I muscle fibers mainly provide energy through the oxidative metabolism, and type II muscle fibers mainly provide energy through the glycolytic metabolism. The composition of muscle fiber is closely related to the activity of related metabolic enzymes. Malate dehydrogenase (MDH) is related to the tricarboxylic acid cycle, and succinate dehydrogenase (SDH) is a key enzyme affecting mitochondrial aerobic respiration, both of which are key enzymes of the aerobic metabolism. It was also found that MDH and SDH activity were positively correlated with the proportion of type I muscle fibers in oxidative muscle fibers, which was consistent with the increase in MDH and SDH content and the proportion of type I muscle fibers in group P in our experiment [41]. LDH is involved in glycolysis, reflecting the level of anaerobic colysis in the body, and its function is to promote pyruvate to produce lactic acid. The reduced LDH content in our experiment indicated a decreased level of glycolysis, which may be associated with the decrease in oxidative muscle fibers in the muscle. According to Bäckhed’s research, the expression levels and protein activities of adenosine 5′-monophosphate-activated protein kinase (AMPK) and carnitine palmitoyltransferase 1 (CPT-1) related to the oxidative metabolism of skeletal muscle mitochondria in sterile mice were higher [42]. In addition, the rapidly induced adipose factor (Fiaf, a major mitochondrial fat regulator) in the intestines of sterile mice could increase the peroxisome-proliferator-activated receptor- γ coactivator (PGC-1 α protein). These results indicate that the energy metabolism efficiency of skeletal muscle in sterile mice decreases after intestinal colonization and that the intestinal flora affects energy balance. Transplanting the intestinal flora of obese pigs into rats could increase the expression of the protein heavy-chain 7 gene in muscle, reduce the expression of protein light-chain 4 in muscle, increase the proportion of slow muscle fibers, and reduce the proportion of fast muscle fibers [43].

## 5. Conclusions

The results of this study show that probiotic supplementation reduces cooking loss and shear force and improves the meat quality of lamb (tenderness). The addition of probiotics alters not only the content of the intestinal metabolite SCFAs but also the expression of muscle fiber genes and the types of muscle fiber in Sunit lamb. Correlation analysis showed that propionic acid and butyric acid were beneficial in promoting the development of oxidative muscle fibers. In conclusion, probiotics-regulated intestinal metabolites can be used as useful biomarkers for regulating muscle fiber types, and this finding provides a theoretical basis for improving the quality of lamb. 

## Figures and Tables

**Table 1:** The primers used for real-time PCR analysis.

Genes	Primer Sequences (5′→3′)	Product Size, bp	Accession NO.
*MyHC I*	F:AAGAACCTGCTGCGGCTG	250	XM_012129251.1
	R:CCAAGATGTGGCACGGCT		
*MyHC IIA*	F:GAGGAACAATCCAATACAAATCTATCT	173	XM_027974884.1
	R:CCCATAGCATCAGGACACGA		
*MyHC IIB*	F:GACAACTCCTCTCGCTTTGG	247	XM_027974883.1
	R:GGACTGTGATCTCCCCTTGA		
*MyHC IIX*	F:GGAGGAACAATCCAATGTCAAC	178	XM_004012706.4
	R:GTCACTTTTTAGCATTTGGATGAGTTA		
*18S rRNA*	F:GTAACCCGTTGAACCCCATT	112	
	R:CCATCCAATCGGTAGTAGCG		

**Table 2:** Effects of probiotics on the muscle fiber properties of Sunit lambs.

	C Group	P Group	*p*-Value
Muscle fiber density (number/mm^2^)	747.43 ± 14.63	828.60 ± 51.45	0.068
Type I muscle fiber			
The number ratio of muscle fibers (%)	9.05 ± 0.81 ^b^	12.08 ± 1.18 ^a^	0.028
The area ratio of muscle fibers (%)	7.53 ± 0.57 ^b^	10.57 ± 0.99 ^a^	0.013
Muscle fiber diameter (μm)	34.54 ± 0.84	37.07 ± 1.81	0.164
Muscle fiber cross-sectional area (μm^2^)	1019.59 ± 43.21	1069.66 ± 84.33	0.568
Type IIA muscle fiber			
The number ratio of muscle fibers (%)	32.23 ± 1.49	31.13 ± 1.65	0.666
The area ratio of muscle fibers (%)	37.26 ± 2.41	37.15 ± 1.70	0.977
Muscle fiber diameter (μm)	42.46 ± 0.75	43.48 ± 1.44	0.496
Muscle fiber cross-sectional area (μm^2^)	1574.39 ± 49.76	1476.37 ± 107.16	0.353
Type IIB muscle fiber			
The number ratio of muscle fibers (%)	57.75 ± 1.49	56.79 ± 2.11	0.652
The area ratio of muscle fibers (%)	54.51 ± 2.36	52.29 ± 2.14	0.582
Muscle fiber diameter (μm)	32.20 ± 0.58	33.30 ± 1.11	0.350
Muscle fiber cross-sectional area (μm^2^)	1242.41 ± 32.80	1126.43 ± 58.17	0.083

^a, b^ Significant differences between lambs fed probiotics (P group) and lambs not fed probiotics (C group) (*p* < 0.05).

**Table 3:** Effect of probiotics on colon short-chain fatty acids (mg/100 g).

Short Chain Fatty Acid (μg/g)	C Group	P Group	*p*-Value
Acetic acid	10.28 ± 0.11	10.29 ± 0.09	0.929
Propionic acid	27.11 ± 0.10 ^b^	27.91 ± 0.27 ^a^	0.031
Butyric acid	57.16 ± 0.15 ^b^	58.27 ± 0.41 ^a^	0.043
Valeric acid	27.98 ± 0.34 ^b^	29.33 ± 0.40 ^a^	0.031
Caproic acid	15.97 ± 2.05	15.23 ± 0.26	0.645
Isobutyric acid	9.68 ± 0.09	9.75 ± 0.05	0.467
Isovaleric acid	26.15 ± 0.17	26.36 ± 0.15	0.404

^a, b^ Significant differences between lambs fed probiotics (P group) and lambs not fed probiotics (C group) (*p* < 0.05).

## Data Availability

Data are contained within the article.

## Supplementary Materials

The following supporting information can be downloaded at: https://www.mdpi.com/article/10.3390/ani13040762/s1, Table S1: gDNA Eraser system; Table S2: Reverse transcription system; Table S3: qPCR system.
