# Supplementary material for: Effects of Probiotics Supplementation on the Intestinal Metabolites, Muscle Fiber Properties, and Meat Quality of Sunit Lamb
Source: Animals (Basel). 2023 Feb 20;13(4):762. doi: 10.3390/ani13040762 (PMC9951964; doi:10.3390/ani13040762)
Supplement: Supplementary file 1 [file animals-13-00762-s001.zip › animals-2106903-supplementary.pdf]

Supplementary Materials

Table S1. gDNA Eraser system.

Reagent	Addition（μL）
RNase Free dH_2O	5.0
5×gDNA Eraser Buffer	2.0
gDNA Eraser	1.0
Total RNA（500 ng/μL）	2.0
Total	10.0

Table S2. Reverse transcription system

Reagent	Addition（μL）
RNA obtained from the previous reaction	10.0
RNase Free dH_2O	4.0
5×PrimeScript Buffer 2 (for Real Time)	4.0
RT Primer Mix	1.0
PrimeScript RT Enzyme MIX Ⅰ	1.0
Total	20.0

Table S3. qPCR system

Reagent	Addition（μL）
TB Green Premix Ex Taq Ⅱ (Tli RNaseH Plus) (2×)	12.5
RNase Free dH_2O	8.5
Primer F	1.0
Primer R	1.0
cDNA template (50 ng/ μL)	2.0
Total	25.0
